# Balancing benefits and barriers: Experiences of digital health consultations among older adults and healthcare staff

**DOI:** 10.1177/20552076261432719

**Published:** 2026-03-16

**Authors:** Karin Myrberg, Annica Björkman, Lars-Christer Hydén, Christina Samuelsson

**Affiliations:** 1 Centre for Research and Development, 560570 Uppsala University/Region Gävleborg, Gävle, Sweden; 2 Faculty of Health and Occupational Studies, Department of Caring Sciences, University of Gävle, Gävle, Sweden; 3 Clinical Science Intervention and Technology, 206106 Karolinska Institutet, Stockholm, Sweden

**Keywords:** Digital health consultations, telehealth, digital equity, older adults, rapport

## Abstract

**Objective:**

Despite having high healthcare needs and being suitable candidates for digital health consultations, older adults (OAs) use these practices less commonly, and are more likely to encounter challenges, which reduces digital health equity. The objective of this study was to examine the experiences of OAs and healthcare staff (HS) using and participating in digital consultations.

**Methods:**

A descriptive approach was employed, using both structured and semi-structured interviews with OAs (≥65 years) and HS who had completed a digital health consultation via video or asynchronous chat within the past week. Interview data were analysed with thematic analysis and descriptive statistics.

**Results:**

The descriptive data (*N* = 33 OAs; 13 HS, each reporting on the same 33 consultations) indicated that both OAs and HS gave mainly positive feedback about their recent digital health consultations, while the qualitative data offered a more nuanced perspective. About half of the OAs would choose a digital consultation again for the same health issue, if given the choice. Practical benefits and professional benefits were highlighted, though benefits for care were also noted by both OAs and HS. Barriers were primarily technical, affecting the conversation in various ways. Barriers to care also arose, mainly relating to experiences of deteriorated contact with the HS.

**Conclusion:**

Digital health consultations offer significant practical benefits for both OAs and HS, and sometimes also benefit care. However, persistent technical and relational barriers highlight the need for improved usability and adaptability. Addressing these challenges and supporting individual preferences are essential steps toward increasing accessibility and ensuring equitable access to digital consultations for OAs.

## Background

The use of digital health technologies is one of the main components in the ongoing transformation of healthcare and is expected to improve patients’ access to medical services as well as their independence.^1^ Additionally, the implementation of digital health technologies is expected to increase effectiveness, and policy-makers often emphasize digitalization as an important solution to the challenges faced in health- and eldercare.^2^

Many new digital healthcare services have emerged in the past couple of years, such as video consultations and chat (hereafter referred to as digital health consultations), allowing healthcare staff (HS) to manage patients remotely and enabling monitoring and continuous care.^3^ Previous studies have highlighted that digital consultations as a substitute for physical consultations are particularly suitable for patients with chronic conditions that require many follow-up visits.^4^ Similarly, they can facilitate access for people living far from healthcare facilities, as well as for patients with fatigue or severe illness.^5^ Hence, older adults (OAs) are often one of the main target groups for digital consultations, as they are overrepresented among those with high healthcare needs and account for a large proportion of total healthcare visits.

OAs are increasingly familiar with digital technologies in general,^6^ as well as with digital health tools such as applications for sleep or heart monitoring, or for tracking daily activities.^7^ However, their experiences with digital technology can still vary greatly, as many OAs are among the late adopters of new technological implementations. Additional challenges to using digital consultations among OAs include lower levels of digital literacy, digital anxiety, physical and cognitive deficits and a lack of perceived usefulness.^8^

There is some research highlighting factors that influence OAs’ perspectives on digital consultations. Yusif et al.^9^ conducted a systematic review of factors affecting OAs’ decisions to adopt digital health technologies, identifying privacy as the main concern, followed by trust, lack of functionality or added value, financial cost and ease of use. In a study by Fleicher et al.,^10^ previous technology experience and access were perceived as crucial for OAs using digital consultations. Participants also described healthcare providers’ perceptions that OAs are incapable of using technology as a barrier. In a study^11^ involving 30 OAs with chronic kidney diseases who received digital health consultations during the pandemic, participants appreciated the convenience and the ease of involving their partners. However, many OAs emphasized a loss of connection with the clinician. Additionally, the study included 19 HS, who perceived a lower quality of care due to a loss of interpersonal connection and increased mistrust. In a study by Reyes et al.,^7^ OAs were motivated to use technologies to monitor health issues, with enablers including easy setup and social or organizational influence. Reported barriers included concerns about privacy and accuracy, as well as the perception that digital health consultations are an inadequate substitute for in-person consultations.

Many digital health tools are developed with homogeneous, highly educated and advantaged populations in mind.^12^ Both Swedish and international studies have revealed that those predominantly using digital health consultations are younger, highly educated individuals living in large cities.^13,14^ This may be one of many reasons why the inclusion of OAs aged 65 and above has been relatively sparse in previous research on digital health consultations.^15^ Although there is growing awareness of digital inequity, important knowledge gaps remain regarding feasibility, quality and patient safety, which must be addressed before digital consultations can be fully recommended for OAs on a broad scale.^14^ For these reasons, we undertook the present study to examine the experiences of OAs (≥65 years of age) using digital health consultations via video or asynchronous chat. Additionally, we investigated the experiences of the HS who interacted with these patients. By adopting what is, to our knowledge, a new perspective, identifying the benefits and barriers faced by both groups in relation to the same consultations, we aim to gather insights to improve digital health consultations for OAs.

## Methods and material

This research primarily adopts a descriptive approach and is based on structured and semi-structured interviews. The reporting of data in this study adhered to the standards for reporting qualitative research by using the COREQ checklist^16^ (see Appendix 1 for details).

The study was conducted within the publicly financed healthcare system of a medium-sized Swedish healthcare region, with data collected between April 2023 and December 2023. In 2022, a proprietary digital health platform was launched to enhance the accessibility and efficiency of healthcare services for the region's residents. The platform offers possibilities for triage, video consultations and two-way asynchronous chats with healthcare professionals. It can be accessed directly via smartphone or tablet, or through a web browser (tablet-sized format). When patients are scheduled for a meeting, they can join the video consultation either by logging into the system directly or by following a link in a text message, both methods requiring secure identification. The application is not linked with the electronic health record.

A stance within Swedish healthcare is that digital consultations should be used ‘where possible and desirable’.^1^ Although the pandemic accelerated the use of digital consultations, data from the region's records indicate that the number of completed digital consultations involving OAs remained very limited, comprising approximately 0.2% of all consultations involving persons aged 65 years and older in 2024.^17^ Data further reveal that the vast majority of HS only sporadically offered digital consultations to OAs, with dieticians as an exception. Unlike most units within the organization, dieticians have continued to maintain these digital practices and now offer most outpatient care patients digital consultations.

### Recruitment and participants

The inclusion criteria for the OAs were: participants aged ≥65 years, who were scheduled for a digital outpatient consultation with a HS, either by video or asynchronous chat. The recruitment procedure naturally excluded OAs who were not considered eligible for a digital consultation based on the HS assessment, including OAs in need of an interpreter or those with dementia. Using the organization's out-data system, we identified a small number of HS who regularly conducted digital consultations with OAs. Using purposive sampling, the HS were contacted and invited to participate in the study. Additionally, snowball sampling was employed, with interviewed HS suggesting colleagues who also worked with digital health consultations. All HS received written information before providing consent. The HS were then asked to keep track of OAs scheduled for digital consultations, resulting in a consecutive sampling method for the OAs. The HS then approached patients for permission to have a researcher contact them with study information. OAs who agreed were sent written materials (invitation, study information and consent form) and were subsequently contacted by KM by telephone for further details. From the outset, it was clearly emphasized that the interviews would focus on the OA's experiences with digital consultations, their recent contact and associated factors, rather than on their health problems or the content of the care itself. By submitting their written consent, participants agreed to take part in the study. None of the OAs wo received the written information declined participation, resulting in 33 OAs (17 women and 16 men) included in the study (Table 1). Their ages ranged from 65 to 88 years (mean 71.5, SD 5.5). The vast majority (69%) had completed a digital health consultation with a dietician during the past week, while the remaining OAs had seen a speech-language pathologist, a registered nurse or physiotherapist. Ten OAs conducted their digital consultation using a computer. Thirteen participants lived more than 20 km from a healthcare facility. In all 33 cases, the initiative for a digital consultation was taken by the healthcare organization. Additional information is provided in Table 1. Participants were recruited until a reasonable range of experiences relevant to the study's aim had been captured, thereby ensuring that the data were adequate to address our research questions.

**Table 1.** Participant detail: OAs, *N* = 33.

Characteristics	*N*	%
Age span		
65–69	15	46
70–74	9	27
75–79	7	21
85–89	2	6
Gender		
Woman	17	52
Man	16	48
Consultation with healthcare profession		
Dietician	23	70
Speech-language pathologist	4	12
Physiotherapist	2	6
Nurse	4	12
Reason for consultation		
Diabetes	10	30
Obesity	8	24
Dysarthria	3	9
Malnutrition	2	6
Cholesterol	3	9
Others	7	21
Device used for consultation		
Smartphone	21	64
Computer	10	30
Tablet	2	6
Highest level of education		
Secondary school	12	36
Upper secondary school	13	39
University	8	24
Distance to health services (km)		
0–9	10	30
10–19	10	30
20–29	1	3
30–39	2	6
40–49	3	9
50–99	5	15
>100	2	6

OA: older adult. Note: percentages may not sum up to 100% due to rounding.

In total, 13 HS, all women, were enrolled in the study, each with at least 2 years of experience in digital health consultations. The majority conducted digital consultations several times a week or more, and three of them worked exclusively from home. For more details, see Table 2.

**Table 2.** Participant detail: HS, *N* = 13.

Characteristics	*N*	
Occupation		
Dietician	7	
Speech-language pathologist	2	
Nurse	1	
Physiotherapist	2	
Gender		
Woman	13	
Primary workplace setting		
Healthcare setting	9	
Home setting	4	
Frequency of digital consultations		
Only digital consultations	3	
Every day	2	
Several times a week	4	
Several times a month	2	
Once a month or less	2	

HS: healthcare staff.

### Interviews

Within 1 week of their digital consultation, the participating OAs and HS were contacted by telephone by KM, an experienced interviewer. Participants were again informed about the study's purpose, introduced to the interviewer and provided with her credentials. None of the researchers had any prior relationship with the participants. All OAs and HS took part in individual interviews that combined structured and semi-structured questions. The structured questions consisted of close-ended yes/no choices or Likert-scale items designed to capture the participants’ experiences. An example of a close-ended question was: ‘As a whole, how did you find the experience of having this appointment by video/chat?’ The semi-structured part included open-ended questions about the participants’ recent experiences, their views on digital consultations in general and specifically for OAs. For instance, an open-ended question was: ‘Can you please tell me about your experiences of your recent digital consultation?’ Furthermore, to encourage participants to elaborate on their responses, probing questions such as ‘Can you give an example?’ and ‘How did you feel then?’ were used. Socio-demographic information regarding age, reason for contact, education and distance to healthcare was also collected (see Appendices 2 and 3 for interview questions). All interviews were conducted via speakerphone, recorded with a portable audio recorder and supplemented by field notes taken during the conversations.

The rationale behind the chosen methodology was its flexibility and the aim to achieve a comprehensive understanding. The close-ended questions were useful for gathering specific, consistent information across participants, while the open-ended questions allowed for rich descriptions and deeper exploration of the fixed answers. Previous studies have shown that patients’ evaluations of healthcare, whenrated on Likert-type scales, tend to be more positive than their verbal responses to open-ended questions.^18^ This underscores the importance of using multiple types of questions to fully capture the ‘voice’ of patients in relation to digital health consultations.

The procedures were piloted in advance with one OA and one HS. The interviews with OAs lasted between 45 and 60 min, while the interviews with HS lasted around 30 min. Participants were free to skip any question or end the interview at any time. Survey results were summarized in a form during the discussions.

### Analyses

Close-ended responses from the OAs and HS were summarized and analysed using descriptive statistics in Jamovi. All answers to the open-ended questions, as well as comments related to the close-ended questions, were transcribed verbatim. We used thematic analysis, a qualitative approach for identifying, analysing and reporting patterns within data, following Braun and Clarke's six-phase method.^19^ Initially, KM, CS and LCH conducted a thorough reading of the transcripts, noting patterns of interest and becoming familiar with the content (Phase 1). During this phase, initial codes were developed to capture as many themes as possible with each transcript coded separately (Phase 2). In the analyses, many of the issues identified were directly related to barriers or enablers expressed in response to adjacent questions, such as those about technology.

Phases 3, 4 and 5 were undertaken simultaneously: candidate themes and sub-themes were identified and visualized using a mind map. At this stage, all coded extracts were reviewed multiple times to ensure they met the inclusion criteria. Following this, KM and AB reviewed the codes to determine their alignment with the proposed themes and sub-themes. Any disagreements regarding the alignment of codes were resolved through discussion until consensus was reached, thereby enhancing the reliability of the analysis through coder triangulation. Afterward, KM continued the analysis by reviewing each theme and sub-theme in relation to the entire data set, searching for relations and variations across cases. The final results were then discussed by all authors (Phase 6), further contributing to the trustworthiness and credibility of the findings. This collaborative and reflexive approach emphasized the researchers’ perspectives and flexibility throughout the process.^20^

### Ethics

All participants gave their written consent to participate and were informed that they had the right to withdraw at any time before the study begun. The participants were assured confidentiality as the interview material was securely stored, with the first author having sole access to it. The study was reviewed and approved by the Swedish Ethical Review Authority in 2022 (No. 2022-05813-01). All methods adhered to relevant guidelines and regulations, including the Declaration of Helsinki.

## Results

### OAs’ and HS’ experiences of digital consultations

#### Descriptive statistics

In this section, we present the results from the close-ended questions (Table 3) as well as the main themes and sub-themes identified from the open-ended questions (Table 4).

**Table 3.** Results (%) from the close-ended questions with OAs, *N* = 33, and HS, *N* = 13, reporting on 33 consultations.

Question	OAs	HS
As a whole, how did you find the experience of having this appointment by video/chat?	Very good: 18 (55%) Fairly good: 6 (18%) Neither good nor bad: 4 (12%) Fairly bad: 4 (12%) Very bad: 1 (3%)	Very good: 23 (70%) Fairly good: 6 (18%) Neither good nor bad: 2 (6%) Fairly bad: 1 (3%) Very bad: 1 (3%)
How did you find the functionality of the technology?	Very good: 15 (45%) Fairly good: 9 (27%) Neither good nor bad: 2 (6%) Fairly bad: 4 (12%) Very bad: 3 (16%)	Very good: 18 (55%) Fairly good: 9 (27%) Neither good nor bad: 1 (3%) Fairly bad: 4 (12%) Very bad: 1 (3%)
Did you manage to connect yourself?	Yes: 20 (61%) No, I needed help: 13 (39%)	-
Did you hear/understand the other person?	Yes: 27 (82%) No: 1 (3%) Partly: 5 (15%)	Yes: 27 (82%) No: 1 (3%) Partly: 5 (15%)
Do you feel that you were able to express what you wanted to say?	Yes: 28 (85%) No: 2 (6%) Partly: 3 (9%)	Yes: 31 (94%) No: 1 (3%) Partly: 1 (3%)
Do you think the conversation was affected in any specific way because it was digital?	Yes: 13 (39%) No: 18 (55%) Maybe: 2 (6%)	Yes: 7 (21%) No: 24 (73%) Partly: 2 (6%)
Would you have done anything differently in a face-to-face consultation?	-	Yes: 12 (36%) No: 15 (45%) Maybe: 6 (18%)
If you could have chosen freely, how would you have preferred this appointment?	Face-to-face: 15 (46%) Video: 15 (46%) Telephone: 1 (3%) Chat: 2 (6%)	-
Do you have previous experience with digital healthcare consultations?	Yes: 10 (30%) No: 23 (70%)	-
If yes to the previous question, has the previous contact been with the same healthcare provider as this appointment?	Yes: 6 (60%) No: 4 (40%)	-
Would you describe yourself as a digital person?	Yes: 14 (42%) No: 6 (18%) Partly: 13 (39%)	-

HS: healthcare staff; OA: older adult. Note: percentages may not sum up to 100% due to rounding.

**Table 4.** Overview of themes and sub-themes from the thematic analysis of open-ended questions with OAs and HS.

Main themes	Sub-themes (OAs and HS)	Sub-themes (OAs only)	Sub-themes (HS only)
Perceived benefits	• Practical benefits • Benefits for the quality of care		• Benefits for the profession
Perceived barriers	• Practical barriers • Barriers to the quality of care • Prejudices about OA's technical skills	• Worries and scepticism towards a new system	
Standardization vs. personalization		• Possibility to determine when digital healthcare is suitable (empowerment)	• Suitability for various healthcare contacts
Recommendations for improvement	• Improvements for care • Technical recommendations	• Personalized support	

HS: healthcare staff; OA: older adult.

Results from the close-ended questions reveal that the responses provided by OAs and HS were quite similar. Despite it being the first digital consultation for a majority (23; 70%) of the OAs, 24 rated their overall experience of the recent digital consultation as ‘very good’ or ‘fairly good’. Among the HS, this rating was given in 29 (88%) out of the 33 cases. Seven (29%) of the OAs experienced the technology function as ‘very bad’ or ‘fairly bad’, whereas the same was expressed by the HS in five of the 33 cases (15%). Thirteen (39%) of the OAs needed help connecting to the meeting. Both the OAs and HS were unanimous in their opinions about whether they could hear the other party in the conversation. The HS reported that in 18 cases (55%), they would definitely or possibly have done something differently if the consultations had been conducted face-to-face. In turn, more than a half (52%) of the OAs indicated that they would choose a digital consultation for this appointment again if given the option. For full details, see Table 3.

#### Thematic analysis

Through thematic analysis, four overarching themes emerged: *perceived benefits, perceived barriers, standardization vs. personalization* and *recommendations for improvement*. Seven sub-themes were identified in responses from both OAs and HS, while three sub-themes were unique to OAs and two were unique to HS. An overview of themes and sub-themes is presented in Table 4.

#### Perceived benefits

Overall, the OAs and the HS had positive views on their recent consultations. The three sub-themes within the *perceived benefits* category, *practical benefits*, *benefits for the quality of care* and *benefits for the profession*, illustrate the opportunities that digital consultations offer to both groups.

Seven of the OAs praised the practical advantages of video consultations. They described the convenience of not having to drive, search for parking or venture out in cold weather. One participant mentioned difficulties with walking, highlighting the benefit of not having to leave home for a meeting. Additionally, two OAs noted the financial savings, particularly important given their low pensions, associated with not having to drive to face-to-face appointments:

It's good, living like this in the countryside, that you don't have to travel. There are no buses where I live, and when you think about gasoline costing 20 SEK per litre, and then having to drive eighty kilometres. That's a lot for me. (OA2)

When it comes to the HS, the practical benefits mentioned were primarily related to improvements in their work environment. Eight of the HS mentioned the newfound possibility of remote work, which had not been available before the pandemic:

It's perfect that I’m able to work from home. There are no dieticians in primary care in the Region I live in. Now I can work with what I want without having to move. (HS1)

Although 10 among the OAs expressed some issues with technology, functionality was also described as a benefit by one:

The technology works perfectly. You just log in at the appointed time, and you receive a reminder SMS beforehand. Then you enter a queue if there are a few minutes left. (OA17)

Another practical benefit mentioned by seven HS was that they experienced digital consultations as more effective and straightforward compared to face-to-face consultations. Two HS noted a positive trend in a reduction of ‘no-shows’ for digital consultations. Two others highlighted not having to fetch patients from the waiting room as a reduction of workload. Additionally, less time spent assisting or helping colleagues when working from home were mentioned. Four HS noted that there is less small talk during digital consultations, making it easier to address the topic directly and resulting in shorter appointments:

The digital consultations tend to be somewhat more efficient and often shorter. This can be due to less small talk, and the conversation is more direct. (HS4)

In terms of benefits for the quality of care, three OAs mentioned as an advantage that it was easier to listen and get the message from the HS while sitting relaxed in their own home:

I definitely think this was a better conversation. I could sit at my table, I had my things, papers, notebook, and pen ready. (OA7)

It was expressed by three other OAs that they preferred the video consultation over a traditional telephone visit, which is rather common as for follow-up appointments:

It's way better than calling, it feels credible when you see each other and know who you are dealing with. You can tell that the person is really listening, she is not reading papers or sitting at the computer at the same time. You take in more of what she says, and it becomes easier to follow her advice. (OA32)

The ability to demonstrate information materials on screen, as well as send them directly, was identified as a benefit by both OAs and HS. Furthermore, the possibility of involving a family member in the consultation was described as advantageous:

The difference is that the patient received several prescription links directly within the program. I can also see if the patient has accessed these. Otherwise, I would have just given the links on a piece of paper or something. (HS4)

The benefits for the profession mentioned by all seven dietitians were new opportunities to work in various parts of Sweden, higher salaries and a newfound sense of freedom, as there are now multiple potential employers in their local areas:

Telehealth also provides our profession with a ‘fuck-off’ capital, if you excuse the language. Previously, the Region has been the only employer in the county. Now, more Regions are hiring digital dieticians and there is an opportunity to change jobs without having to move. (HS4)

#### Perceived barriers

Barriers with their recent digital consultation were mentioned by 14 OAs in the open-ended responses. The five sub-themes reflected in the *perceived barriers* category relate to *practical barriers, barriers to care, worries and scepticism towards a new system* and *prejudices about OAs’ technical skills.*

Among the practical barriers, 10 OAs mentioned various issues with technology. There was a widespread desire for a more user-friendly application for digital consultations than the platform currently provided by the healthcare organization. Connection problems and difficulties hearing were also mentioned. Four OAs described a lack of clarity with the instructions and the link sent out for connection:

The technology didn’t quite cooperate. I was supposed to do it via the computer somehow, which they said was better, but then an SMS came to my phone and that was what I had to click on. So it ended up being on the phone anyway, and it worked at first, but then there were issues with the connection or something, it got all scrambled and noisy. (OA29)

Among the HS, it is discussed that many OAs conduct the meetings via their smartphone, even though it is recommended to use a computer. It was mentioned that this differs significantly as compared to their younger patients. This results in problems with demonstrating on-screen materials, as it can be difficult to see on a small display. Eight HS mentioned OAs filming their ceiling, the top of their face or moving the camera back and forth during the meetings, which complicates communication. One of the HS (HS2) discussed whether it is appropriate to tell the patient or not but decided against it.

Ten OAs mentioned barriers to care: although they were content with their recent digital consultation, they did not believe it is possible to achieve the same level of contact or connection as compared to face-to-face consultations:

It's a different kind of meeting when you meet in person. You get a different connection thanks to body language, tone of voice and things like that. (OA16)

Five HS described more hands-on barriers to the quality of care in relation to their recent consultation, such as not being able to weigh the patients, the inability to get a full picture of the patient (only seeing their face) and not being able to perform a physical examination or make adjustments to the patient's training equipment when needed. Additionally, HS11, a dietician, mentioned that going out for a face-to-face consultation might be an important activity in itself for some OAs:

A disadvantage for OAs is that many miss the opportunity to get out. This is something that many of those we meet desperately need. (HS11)

A number of worries and scepticism about a new system were mentioned. Thirteen OAs needed help connecting to their meeting. This is further illustrated in the open-ended responses, where seven OAs described receiving assistance from their children or grandchildren when downloading the application. Additionally, some participants mentioned having a relative nearby for support during the meeting:

My husband sat with me for support, but he doesn't really know anything about it, so I'm not sure what good it did. I got help from the dietician later, it will be easier next time. (OA29)

Approximately 40% (14) of the OAs described themselves as ‘digital persons’, while another 40% (13) considered themselves to be ‘partly digital’. In the interviews, many OAs described frequent use of social media such as Facebook, reading the news and face-timing with their grandchildren. However, some OAs expressed their own uncertainty, and even more described a belief that their peers were generally reluctant towards technology:

I think many people are skeptical, like I was at first, thinking, ‘No way, I don't want to be a part of that.’ Especially if they haven't tried it. (OA20)

In relation to the use of digital consultations within the organization, some HS described a common worry and scepticism about the new work procedures and systems among their healthcare colleagues. Additionally, some HS mentioned the convenience of their non-digital colleagues’ current routines, along with a reluctance to change:

It is the healthcare staff who are holding back, I see it in my colleagues. It is about some not being willing to change and their own lack of knowledge. We aren't the youngest ladies working with diabetes. (HS9)

The HS consistently cited their colleagues’ prejudices regarding OAs’ technical skills as a reason for the limited number of digital consultations offered to this group. This sentiment was also experienced by one of the OAs:

When I was asked by a physiotherapist if I wanted to meet with a dietician, it was more like ‘well, if you can manage to connect, you can meet with a dietitian digitally’. I didn't say anything, but it felt kind of like they thought I was from the Stone Age or something. (OA8)

#### Standardization vs. personalization

The individual preferences and needs of the OAs were reflected by the participants in the following sub-themes: *the possibility to determine when digital healthcare is suitable*, and *suitability for various healthcare contacts*.

Eleven OAs expressed that although they were positive about having a digital consultation, they would not consider having a GP consultation or a more ‘serious’ appointment digitally. One OA highlighted that having a digital consultation for her particular health issue was much appreciated:

It felt very nice to avoid having a face-to-face consultation; I find it a bit difficult to tackle my overweight. It was very convenient; I just sat on the couch and clicked the link. (OA8)

If they had been able to choose freely the next time, 52% of the OAs would opt for a digital consultation. One HS discussed these empowerment issues and the problematic fact that currently, their patients don not really have a choice:

A disadvantage for the patient might be that they don't really get to choose. The basic idea is that they should be able to choose, but often I think they just receive a summons for a digital visit or we try to ‘persuade’ them. (HS10)

The HS were also consistent in their descriptions, noting that digital consultations are not suitable for OAs who need an interpreter, have language or communication difficulties or have cognitive disabilities.

#### Recommendations for improvement

We identified a number of recommendations and advice from OAs towards healthcare, as well as from HS to their colleagues, resulting in three sub-themes:

First, *personalized support*. Eight OAs emphasized that it would have been beneficial to receive clearer instructions or a demonstration in advance from the HS, preferably by telephone or a face-to-face appointment Additionally, two OAs suggested that their local health centres should offer such education opportunities and support. Two other OAs recommended that healthcare providers should not assume that all patients have the necessary equipment available:

Healthcare needs to remember that it's not a given that every older person has the necessary technology, like headphones and such. (P18)

Second, *technical recommendations* are discussed by several of the OAs that the application itself is not user-friendly, and particularly not for older persons. One OA noted that it should be a general support number to call when one is experiencing troubles in the application.

Third, *improvements for care*. The only nurse enrolled discussed the advantages of her self-developed routine for patients with diabetes, combining face-to-face (involving physical examinations) with digital consultations, and suggested that other colleagues try it. Another practical piece of advice was provided by three HS emphasizing the importance of intentionally including small talk during digital consultations, as it is important for the clinical relationship:

Don't forget the small talk! I always make sure to engage in a bit of small talk at the beginning of each digital appointment. It's really important for many of our patients and can be easy to overlook compared to a face-to-face appointment. (HS2)

## Discussion

The objective of this study was to examine the experiences of both OAs and HS participating in digital consultations. Few studies have explored OAs’ experiences of digital consultations, but to our knowledge, this is the first study to also include the perspectives of HS from the other side of the same consultation. Overall, both OAs and HS reported positive experiences with their recent consultation on the close-ended questions. However, the detailed analysis highlighted not only practical benefits, but also barriers, including those related to the quality of care, which are important to consider and address within healthcare.

The practical benefits described by the OAs, such as convenience, saving time and energy and reducing travel, are especially relevant for those with chronic conditions, financial constraints, or who live far from health services, as also noted in previous studies.^5,4,21^ For OAs residing in rural areas with long distances to healthcare, offering digital consultations may be particularly important.

Practical barriers experienced by OAs were primarily related to technology, such as application usefulness, connectivity or hearing issues. Thirteen of the 33 OAs needed assistance to connect to the digital consultation, and problems with navigating the application, maintaining the connection or viewing on-screen material were also mentioned. Greenhalgh and colleagues^22^ have highlighted the need for healthcare organizations to proactively improve digital inclusion. It is also evident that many providers of digital healthcare services assume that the user has a high ability to read, write and draw conclusions from phenomena that are sometimes implicit.^23^ Hence, using digital tools is not always uncomplicated for OAs. Without a focus on equity and consideration for a broad range of end users, there is a risk that the digital transformation of healthcare, while aiming for greater equity, could actually have the opposite effect. In the setting of this study, patients are advised to use a computer and headphones for digital consultations; however, only 10 OAs followed this recommendation, with the remaining 23 using smartphones or tablets. Some OAs reported not owning a computer or headphones, instead relying on smartphones for their digital needs. The equipment issue was also raised by HS, who described OAs filming their ceiling or walking around during consultations. This raises questions about the strength and feasibility of current recommendations, and whether healthcare organizations should provide equipment for borrowing to ensure quality of care is not compromised.

Previous discussions have highlighted that subtle signs might be overlooked during digital consultations.^10^ Interestingly, better equipment or clearer guidelines on how to use it could potentially resolve many of the concerns raised by OAs, including worries, scepticism towards the system and perceived barriers to the quality of care. These barriers are often related to difficulties in building rapport with HS, leading to a sense of diminished contact compared to face-to-face consultations. In a conceptual analysis of therapeutic relational connection within telehealth, Duffy et al.^24^ concluded that alternative approaches are needed to establish strong therapeutic relationships in digital settings, and that HS require specific guidelines and education for this purpose. The concept of ‘digital empathy’, which involves adapting traditional empathetic skills to digital consultations, has been proposed as such an approach.^25^ To foster digital empathy, small talk is considered a prerequisite.^26^ Notably, three of the dieticians in the present study emphasized the importance of incorporating small talk into consultations, an aspect that could be beneficial to include in organizational guidelines and training.

Among the HS, all 13 participants appreciated the practical benefits of digital consultations, particularly improvements in their work environment, such as the opportunity to work from home. They consistently found digital consultations to be time-saving, often attributing this to a reduction in small talk, a factor previously noted in digital consultations.^27^ Thus, the general notion of digital consultations as time-saving and effective^2^ aligns well with the experiences of the HS in this study. High workload and turnover among HS are challenges faced by many countries,^28^ making it important to consider the impact that a shift toward more digital consultations might have on these issues. However, it should also be noted that benefits for the work environment and the profession do not automatically translate to benefits for patients.

In the data from the present study, OAs described the suitability of digital consultations for specific healthcare contacts, and about half indicated that they would choose a digital consultation again for the same health issue, likely due to the benefits identified. Detailed analyses revealed that this willingness is related to the type of care, with hesitancy to use digital visits for ‘serious’ or ‘real’ healthcare needs. HS noted that certain individuals, such as those with communication problems or cognitive impairments, are less capable to engage in digital consultations. However, it is debatable whether digital consultations should also be offered to these patient groups to empower them to make their own health decisions, in line with the principles of person-centred care.^29^

It is evident that prejudices about OAs’ digital skills may contribute to the low number of digital consultations offered to OAs within the organization where this study was conducted. Previous research has shown that HS with higher levels of ageism are more likely to perceive OAs as incapable of using digital technology for healthcare.^30^ Interestingly, this type of ageism may also be present among OAs themselves, as several participants in the present study mentioned that many of their peers are sceptical and not digitally engaged.

Greenhalgh and colleagues^22^ highlight a mismatch between policy visions for digital consultations and practical reality. While policymakers describe digital consultations as efficient, safe and accessible, contradictions and tensions remain. For example, there are questions about the appropriate level of support from patients’ relatives and friends, the impact on relational continuity, when to include a physical examination and how much time should be allocated to digital queries. It is crucial that digital health consultations are tailored to meet the needs of OAs. The present study emphasizes that improvements can often be achieved through relatively simple measures. These include providing technical support and education on effective consultation practices for OAs, establishing clinical routines for digital consultations across patient groups, and encouraging digital empathy, for example by incorporating small talk. Careful examination of what constitutes a positive healthcare experience can inform the design of care models that better address OAs’ needs and ultimately lead to improved care experiences.

### Strengths and limitations of the study

As expected, OAs in this study described both positive and negative experiences with digital consultations. While most OAs reported satisfaction on Likert-scale questions, their open-ended responses revealed a more nuanced perspective, including barriers and suggestions for improvement. This aligns with previous findings that Likert-type scales may produce positively biased satisfaction scores, whereas open-ended questions provide deeper insights.^18^ The use of both structured and semi-structured questions is thus a strength, as it allows for a more comprehensive understanding of patient experiences. The observed discrepancies may indicate that while OAs are generally positive about their care, open-ended questions encourage more nuanced or critical reflections. It is also possible that they are reluctant to give an overall negative evaluation but feel more comfortable voicing specific concerns when prompted by open-ended questions.

A limitation of the study is the wide age range among OAs (mean 71.5), with most participants in the younger segment. While this broad inclusion was necessary due to the limited number of digital consultations offered to OAs, it could mean diverse experiences within the group. Additionally, for nearly 70% of OAs, this was their first digital consultation, so their responses may reflect initial impressions rather than established opinions. Another limitation is the purposive sampling of HS who were already actively engaged in digital consultations, which may have led to an overrepresentation of those more interested in or positive towards such consultations. The lack of diversity among the HS is also a limitation, as the group was composed entirely of females and predominantly of dieticians. This homogeneity may have influenced findings and does not capture the perspectives of male HS or other professional groups, such as physicians. However, the sample does reflect the actual distribution of digital consultations within the organization.

Limited participant numbers precluded certain analyses, such as stratification by age, education or distance to health services, and the sample is not statistically representative, restricting generalizability. Non-Swedish speakers and cognitively impaired individuals were excluded, as they were not eligible for digital consultations, decisions often based on HS judgment rather than standardized criteria. Lastly, interviews were conducted by a single researcher, which ensured consistency, though all four authors contributed to data analysis, enhancing dependability.

## Conclusions

This interview study provides valuable insights into OAs’ and HS's experiences of digital health consultations. The results offer important guidance for healthcare organizations seeking to develop effective digital consultation procedures in general, and for OAs in particular. The findings reinforce the importance of tailoring digital consultations to meet the individual needs of OAs and suggest several recommendations for improvements. Beyond technological usability, addressing practical barriers, such as access and familiarity with equipment, as well as providing technical support and targeted training for both OAs and HS, is essential. The study also underscores the need for digital inclusion policies and infrastructure to ensure equitable access. Addressing barriers to care, such as challenges in building therapeutic relationships and the risk of excluding certain patient groups, is essential for optimizing the quality of care. These findings can inform ongoing and future development of digital health services for OAs, and more research is needed to identify best practices for this growing modality of care.

## Supplemental Material

sj-docx-1-dhj-10.1177_20552076261432719 - Supplemental material for Balancing benefits and barriers: Experiences of digital health consultations among older adults and healthcare staff Supplemental material, sj-docx-1-dhj-10.1177_20552076261432719 for Balancing benefits and barriers: Experiences of digital health consultations among older adults and healthcare staff by Karin Myrberg, Annica Björkman, Lars-Christer Hydén and Christina Samuelsson in DIGITAL HEALTH

sj-docx-2-dhj-10.1177_20552076261432719 - Supplemental material for Balancing benefits and barriers: Experiences of digital health consultations among older adults and healthcare staff Supplemental material, sj-docx-2-dhj-10.1177_20552076261432719 for Balancing benefits and barriers: Experiences of digital health consultations among older adults and healthcare staff by Karin Myrberg, Annica Björkman, Lars-Christer Hydén and Christina Samuelsson in DIGITAL HEALTH

sj-docx-3-dhj-10.1177_20552076261432719 - Supplemental material for Balancing benefits and barriers: Experiences of digital health consultations among older adults and healthcare staff Supplemental material, sj-docx-3-dhj-10.1177_20552076261432719 for Balancing benefits and barriers: Experiences of digital health consultations among older adults and healthcare staff by Karin Myrberg, Annica Björkman, Lars-Christer Hydén and Christina Samuelsson in DIGITAL HEALTH
