# Supplementary material for: Balancing benefits and barriers: Experiences of digital health consultations among older adults and healthcare staff
Source: Digit Health. 2026 Mar 16;12:20552076261432719. doi: 10.1177/20552076261432719 (PMC13009575; doi:10.1177/20552076261432719)
Supplement: sj-docx-1-dhj-10.1177_20552076261432719 - Supplemental material for Balancing benefits and barriers: Experiences of digital health consultations among older adults and healthcare staff [file sj-docx-1-dhj-10.1177_20552076261432719.docx]

| **Item** | **Guide questions/description** | **Reported on page (#)** |
| --- | --- | --- |
| **Domain 1: Research team and reflexivity** |  |  |
| 1. Interviewer/facilitator | Which author/s conducted the interview? | 7 |
| 1. Credentials | What were the researchers’ credentials? | 7 |
| 1. Occupation | What was their occupation at the time of the study? | 7 |
| 1. Gender | Was the researcher male or female? | 7 |
| 1. Experience and training | What experience or training did the researcher have? | 7 |
| 1. Relationship with participants established | Was a relationship established prior to study commencement? | No |
| 1. Participant knowledge of the interviewer | What did the participants know about the researcher? | 7 |
| 1. Interviewer characteristics | What characteristics were reported about the interviewer/facilitator? | 7 |
| **Domain 2: Study Design** |  |  |
| 1. Methodological orientation and theory | What methodology orientation was started to underpin the study? | 4-7 |
| 1. Sampling | How were participants selected? | 5 (purposive sampling of healthcare staff, and consecutive sampling of older adults). |
| 1. Method of approach | How were participants approached? | 5 |
| 1. Sample size | How many participants were in the study? | 6 |
| 1. Non-participation | How many people refused to participate or dropped out? Reasons? | None |
| 1. Setting of data collection | Where was the data collected? | 4 |
| 1. Presence of non-participants | Was anyone else present besides the participants and researchers? | No |
| 1. Description of sample | What are the important characteristics of the sample? | 6 and 7 |
| 1. Interview guide | Were questions, prompts, guides provided by the authors? | See Appendix 2 and 3. |
| 1. Repeat interviews | Were repeat interviews carried out? | No |
| 1. Audio/visual recording | Did the research use audio or visual recording to collect the data? | 8 |
| 1. Field notes | Were field notes made during and/or after the interview? | 8 |
| 1. Duration | What was the duration of the interviews? | 8 |
| 1. Data saturation | Was data saturation discussed? | 6 |
| 1. Transcripts returned | Were transcripts returned to participants for comment and/or correction? | No |
| **Domain 3: analysis and findings** |  |  |
| 1. Number of data coders | How many data coders coded the data? | 9 |
| 1. Description of the coding tree | Did authors provide a description of the coding tree? | No |
| 1. Derivation of themes | Were themes identified in advance or derived from the data? | 9 |
| 1. Software | What software, if applicable, was used to manage the data? | NA |
| 1. Participant checking | Did participants provide feedback on the findings? | No |
| 1. Quotations presented | Were participant quotations presented to illustrate the themes/findings? Was each quotation identified? | 12-18 |
| 1. Data and findings consistent | Was there consistency between the data presented and the findings? | 12-18 |
| 1. Clarity of major themes | Were major themes clearly presented in the findings? | 12-18 |
| 1. Clarity of minor themes | Is there a description of diverse cases or discussion of minor themes? | 12-18 |
