# Supplementary material for: Balancing benefits and barriers: Experiences of digital health consultations among older adults and healthcare staff
Source: Digit Health. 2026 Mar 16;12:20552076261432719. doi: 10.1177/20552076261432719 (PMC13009575; doi:10.1177/20552076261432719)
Supplement: sj-docx-2-dhj-10.1177_20552076261432719 - Supplemental material for Balancing benefits and barriers: Experiences of digital health consultations among older adults and healthcare staff [file sj-docx-2-dhj-10.1177_20552076261432719.docx]

**Interview guide: Older Adults**

1. As a whole, how did you find the experience of having this appointment by video/chat? (Very good, Fairly good, Neither good nor bad, Fairly bad, Very bad)
2. How did you find the functionality of the technology? (Very good, Fairly good, Neither good nor bad, Fairly bad, Very bad)
3. Did you manage to connect yourself? (Yes, No I needed help)
4. Did you hear/understand the other person? (Yes, No, Partly)
5. Do you feel that you were able to express what you wanted to say? (Yes, No, Partly)
6. Do you think the conversation was affected in any specific way because it was digital? (Yes, No, Maybe)
7. If you could have chosen freely, how would you have preferred this appointment? (Face-to-face, Video, Telephone, Chat)
8. Do you have previous experience with digital healthcare consultations? (Yes, No)
9. If yes to the previous question, has the previous contact been with the same healthcare provider as this appointment? (Yes, No)
10. Would you describe yourself as a digital person? (Yes, No, Partly)
11. Can you describe your overall experience with the digital consultation?
12. What are your perspectives on digital consultations in general and for OAs in particular?
13. Do you have any additional comments, or want to add something?
